# Microbial Biomarkers for Lung Cancer: Current Understandings and Limitations

**DOI:** 10.3390/jcm11247298

**Published:** 2022-12-08

**Authors:** Jiawen Huang, Juan Huang

**Affiliations:** Department of Hematology, Sichuan Academy of Medical Sciences and Sichuan Provincial People’s Hospital, University of Electronic Science and Technology of China, Chengdu 610056, China

**Keywords:** lung cancer, microbiome, biomarker, respiratory tract, lung dysbiosis

## Abstract

As our “hidden organ”, microbes widely co-exist at various sites on the human body. These microbes are collectively referred to as the microbiome. A considerable number of studies have already proven that the microbiome has significant impacts on human health and disease progression, including cancers. The recent discovery of cancer-specific microbiomes renders these cancer-associated microbes as potential biomarkers and therapeutic targets. While at low biomass levels, the lung microbiome still dramatically influences the initiation, progression and treatment of lung cancers. However, research on lung cancer-associated microbiomes is emerging, and most profiling studies are performed within three years. Unfortunately, there are substantial inconsistencies across these studies. Variations in microbial diversity were observed, and different microbial biomarkers for lung cancer have been proposed. In this review, we summarized the current findings of lung cancer microbiome studies and attempt to explain the potential reasons for the dissimilarities. Other than lung microbiomes, oral and airway microbiomes are highly related to lung microbiomes and are therefore included as well. In addition, several lung cancer-associated bacterial genera have been detected by different independent studies. These bacterial genera may not be perfect biomarkers, but they still serve as promising risk factors for lung cancers and show great prognostic value.

## 1. Introduction

A vast number of microorganisms, or microbes, colonize various human body sites, such as the gut, skin, mouth and respiratory tract. Such microorganisms include (but are not limited to) bacteria, yeasts, fungi and viruses, and they are collectively known as the microbiome or microbiota. While microbiota refer to microorganisms in a specific environment, the microbiome describes a collection of genomes from all microorganisms in the environment. As this review mainly discusses the genomic profiling of lung cancer-associated microorganisms, the term “microbiome” is used. These microbes are at least as abundant as the somatic cells in an individual [1] and contain far more genetic information than a human genome [2]. Numerous studies have revealed the essential roles of the microbiome in health and disease via the modulation of signaling transduction, metabolism, tissue homeostasis and immune responses [3,4,5,6,7].

It is not surprising that the microbiome influences cancer progression and treatment [8]. For example, *Helicobacter pylori* is a well-known carcinogen contributing to gastric cancer initiation [9]. *Fusobacterium nucleatum* is also closely related to the invasion and metastasis of colorectal cancer [10]. Recently, Nejmen et al. [11] performed a comprehensive analysis of 1526 tumors and paired paracancerous normal tissues across seven tumor types and discovered that different tumor types harbor distinct intratumor microbiome compositions. These intratumor bacteria correlate with the patient’s underlying diseases, status and response to treatment, suggesting the use of tumor-resident microbiomes as diagnostic biomarkers. Moreover, a recent finding suggests that even low abundant intratumor microbes can reshape the cytoskeleton and promote the survival of circulating tumor cells, leading to tumor metastasis [12]. This indicates that the tumor-resident microbiome is not only a result of tumor formation, but they also actively contribute to cancer development. Furthermore, recurrent HLA-I-bound peptides derived from tumor-residing intracellular bacteria were identified on tumor cells in melanoma patients, and they can induce the activation of tumor-infiltrating T cells [13]. Such findings strongly support targeting specific intratumor bacteria in anti-tumor immunotherapy.

Lung cancer is currently the global leading cause of cancer-caused death, claiming 1.80 million lives in 2020 [14]. Given the crucial role of the cancer-resident microbiome in cancer progression and diagnosis, it would be worth investigating the microbiome of lung cancer tissues as well. Such microbiomes can be potential microbial biomarkers of lung cancers and may contribute to tumorigenesis. However, lung tissues are difficult to acquire and it is almost impossible to obtain the control tissues from healthy individuals. Therefore, due to their close relationship (which will be elaborated upon later), many studies also used samples from the mouth or other respiratory tract sites to study the lung cancer-associated microbiome. In this review, we focus on lung cancer-associated microbiome profilings in the mouth and respiratory tract (including the lung). The potential bacterial biomarkers are introduced, and the reasons for dissimilarities across studies are discussed.

## 2. Lung or Tumor-Resident Microbiome and Lung Cancers

While human lungs are constantly exposed to the environment and microbes, healthy lungs were traditionally considered sterile organs (i.e., free of resident bacteria) [15]. However, since the development of culture-independent molecular techniques such as 16s rRNA sequencing, numerous studies revealed many lung-residing microorganisms (i.e., bacteria, viruses, and fungi). In healthy lungs, such lung microbiomes are in low abundance, with a much lower microbial biomass than other body sites [16]. Despite the low microbial burden, the lung microbiome is still paramount for body homeostasis and health, including the modulation of inflammation [17,18] and pathogen or pollutant protection [11,19].

As for lung cancers, the newly emerged spatial meta-transcriptomics revealed that the bacterial load in lung cancer cells is significantly higher than that in the surrounding stromal cells and immune cells [20]. Moreover, a clear decreasing trend in bacterial load was observed from the airways to lung cancer cells to tertiary lymphoid structures and the adjacent normal lung tissues [20] (or upper airways to lower airways and lung [21]). Similarly, oral taxa were identified in bronchoscopy and lobectomy samples, with a higher abundance in bronchoscopy samples [22]. Therefore, lung cancer-resident bacteria likely originate from environmental exposure and the oral/airway microbiome rather than the colonization of gut-derived bacteria from blood (which is another primary source of intratumor bacteria for many cancer types). It should be noted that there are substantial inconsistencies across studies of lung cancer microbiomes (which will be elaborated in Section 2.1 and Section 2.2); the findings concerning bacterial load in lung cancer should be evaluated with caution. Nevertheless, the lung-resident microbiome has great diagnostic and prognostic potential, and the profiling of the lung cancer-resident microbiome is reviewed herein (Table 1).

### 2.1. Few Commonalities across Different Lung Microbiome Profilings

In one of the earliest characterizations of human lung tissue and lung cancer microbiome using a large cohort, Yu et al. [23] analyzed non-malignant lung tissue samples from 165 lung cancer patients and tumor samples from 31 patients. They identified unique microbial communities in the lung, with the dominant phylum *Proteobacteria*. Moreover, the abundance of genus *Thermus* and *Legionella* in paracancerous lung tissues is elevated in patients with advanced stages and metastasis. Similarly, the abundances of *Thermus* and *Ralstonia* differ in different lung cancer subtypes (i.e., lung adenocarcinomas and lung squamous cell carcinoma). Therefore, these bacteria may serve as potential biomarkers for lung cancer prognosis. However, numerous studies following lung cancer-associated microbiome profilings do not provide similar findings. Such dissimilarities occur in the specific tumor-resident bacteria identified and involve the overall microbiome diversity or richness.

**Table 1:** Summary of studies of lung cancer-associated microbiome in the oral cavity and respiratory tract (most recent first).

Study	Sampling Site	Disease	Experiment Design	Sequencing Methods *	Diversity Variations in LC	Microbial Associations or Biomarkers
Yuan [24]	Tumor	LC	RM LC (n = 174) vs. non-RM LC (n = 134)	TCGA	RM LC has similar α-diversity, but reduced richness	*Acidovorax*, *Clostridioides*, *Succinimonas*, *Shewanella*, *Leuconostoc* and *Dickeya* are biomarkers for RM LC
Baranova [25]	Sputum	LUSC	Patients (n = 40) vs. healthy controls (n = 40); all male	16S V3–V4	Decreased β-diversity in LUSC; no changes in α-diversity	*Streptococcus*, *Bacillus*, *Gemella and Haemophilus* are enriched in LUSC patients
Wu [26]	BALF; tumor	LC as GGO	BALF from diseased lung and paired contralateral healthy lung (n = 11); lung GGO and paired adjacent normal tissues (n = 26)	16S V4/16S V3/16S V3–V4/16S V4–V5	No changes in α- and β-diversity	Significantly reduced *Proteobacteria* in LC tissues; In BALF of LC patients: reduced *Rothia*, and increased *Lachnospiraceae*, *Bacteroides uniforms* and *Faecalibacterium prausnitzii*
He [27]	Sputum	NSCLC and/or COPD	patients with NSCLC and COPD (CN, n = 67) vs. NSCLC (n = 9) vs. COPD (n = 14)	16S V3–V4	No significant differences in diversities	In CN patients: reduced *Streptococcus*, *Veillonella*, *Moraxella* and *Actinomyces*; and increased *Neisseria* and *Acinetobacter*
Vogtmann [28]	Oral wash	LC	Patients (n = 1306)	16S V4	Higher α-diversity is associated with lower LC risk	Increased *Streptococcus* and *Peptoniphilus* abundances are associated with increased LC risk, while *Peptostreptococcus*, *Eubacterium yurii* and *Aggregatibacter* are associated with reduced risk
Kim [29]	Tumor	NSCLC	Tumor tissue (n = 162) vs. adjacent normal tissue (n = 54)	16S V4–V5	Reduced diversity as LC progress	Increased *Romboutsia*, *Novosphingobium*, *Acinetobacter* and *Prevotella* in LC Increased *Stenotrophomonas* upon LC relapse
Qian [30]	BALF; airway protected brushing	sMPLC	Patients (n = 8)	16S V3–V4	Increased α-diversity in BALF	*Clostridium*, *Actinobacteria*, *Fusobacterium* and *Rothia* are enriched in the BALF of sMPLC lesions
Chen [19]	Tumor	LC	Tumor tissue (n = 34) vs. adjacent normal tissue (n = 29)	16S	Lower α-diversity and higher β-diversity in LC tissues	In LC: increased *Staphylococcus*, *Capnocytophaga*, *Lachnoanaerobaculum*, *Fusobacterium*, *Oligella*, *Rubellimicrobium*, *Marinococcus Sphingomonas* and *Sphingopyxis*; and decreased *Comamonas* and *Peptococcus*
Masuhiro [31]	BALF	LC	Patients under PD-1 blockade treatment (n = 12)	16S V3–V4	Higher diversity in responders	Responders have higher *Bacteroidetes* and lower *Proteobacteria*
Zhang [32]	BALF; tumor	NSCLC	Patients (n = 6 for BALF; n = 37 for tumor tissues)	Pathogen-targeted sequencing (tumor and 4 BALF); 16S (2 BALF)	Higher diversity in BALF than in tumor	BALF and tumor tissues share *Streptococcus pneumoniae*, *S. crista*, *S. constellatus*, *S. gordonii*, *Prevotella II*, *Haemophilus*, *H. haemolyticus*, *H. influenzae*, *Actinomyces Neesii*, *human herpes virus type 7* and *Neisseria lactose*
Marshall [33]	Epithelial brushing	Pre-cancer	A 10-year follow-up study of 393 patients: with incidence (n = 59), prevalence (n = 21), and no cancer (313)	16S V4	NA	The abundances of *Bacilli*, *Lactobacillales*, *Streptococcus* and *Paenibacillus* are associated with incident LC
Zeng [34]	BALF	NSCLC	LC (n = 46) vs. benign lung disease (n = 29)	16S V3–V4	Increased α-diversity during carcinogenesis and significant changes in β-diversity	Enrichment of phyla (*Firmicutes* and *Bacteroidetes*) and genera (*Streptococcus*, *Prevotella* and *Veillonella*) in NSCLC
Chu [35]	BALF	LC	Patients under PD-1 blockade treatment: responders (n = 19) vs. non-responders (n = 27)	16S V3–V4	Decreased diversity upon treatment	Increased abundance of Fusobacterium is associated with poor anti-PD-1 therapy response
Zhang [36]	Tumor	NSCLC	Patients (n = 53)	Pathogen-targeted sequencing	NA	At advanced stage: increased *Serratia marcescens*, *Actinomyces neesii*, *Enterobacter cloacae* and *Haemophilus parainfluenzae*; and decreased *Staphylococcus haemolyticus* and *Streptococcus crista* Survival prediction: *Haemophilus parainfluenzae*, *Serratia marcescens*, *Acinetobacter jungii* and *Streptococcus constellation* High PD-L1 expression: increased *Acinetobacter jungii*
Huang [37]	Sputum	NSCLC	Patients (n = 85)	16S V3–V4	Decreased α- and β-diversity at advanced stage	Early stage: *Granulicatella* and *Actinobacillus* are enriched Advanced stage: *Actinomyces* is enriched
Roy [38]	Saliva	LUAD	Patients (n = 5) and healthy control (n = 5)	16S V3–V4	No significant changes in α-diversity	Increased *Rothia mucilaginosa*, *Veillonella dispar*, *Prevotella melaninogenica*, *Prevotella pallens*, *Prevotella copri*, *Haemophilus parainfluenzae*, *Neisseria bacilliformis* and *Aggregatibacter segnis* in LUAD
Dong [39]	Tumor	LC	Tumor tissues (n = 118) vs. adjacent normal tissue (n = 123) from 143 patients	16S V3–V4	No significant changes in α-diversity but significant differences in β-diversity	*Massilia*, *Phenylobacterium* and *Pseudoxanthomonas* are enriched in tumor tissue; *Brevibacillus*, *Cupriavidus* and *Anaerococcus* are enriched in normal tissues *Massilia* and *Acidovorax* are associated with TP53 mutation
Jang [40]	BALF	LC	Patients under PD-1 blockade treatment (n = 84)	16S V3–V4	No significant changes in α-diversity and β-diversity	High-PD-L1 group: dominated by *Veillonella dispar*; with reduced *Neisseria* Responders: dominated by *Veillonella dispar* Non-responders: dominated by *Haemophilus influenzae* and *Neisseria perflava*
Boesch [41]	Tumor	AdvancedNSCLC	Tumor tissues (n = 38) vs. adjacent normal tissue (n = 10) from patients with PD-1 blockade treatment	16S V3–V4	Increased diversity is associated with better survival	Gammaproteobacteria correlate with low PD-L1 expression and poor anti-PD-1 blockade treatment outcomes
Lu [42]	Sputum	NSCLC	Patients (n = 87) vs. healthy controls (n = 34)	16S V3–V4	Decreased α-diversity in NSCLC	NSCLC: increased *Haemophilus parainfluenzae* and *Haemophilus influenzae* Distant metastasis: decreased *Capnocytophaga*; and increased *Pseudomonas*, *Coriobacteriaceae* and *Actinomyces*
Chang [43]	Tumor	LC	Patients (n = 49)	16S V4	NA	*Brevundimonas diminuta*, *Acinetobacter radioresistens Enterobacter cloacae*, *Mycobacterium chelonae*, *Mycobacterium franklinii*, *Staphylococcus* sp., *Bacillus megaterium*, *Pseudomonas aeruginosa* and *Rhodococcus erythropolis* are enriched in LC and associated with poor prognosis
Shi [44]	Mouth rinse	LC	Patients (n = 156) vs. healthy control (n = 156)	16S V4	No significant changes in α-diversity and β-diversity	The abundances of families *Lachnospiraceae*, *Peptostreptococcaceae*, *Erysipelotrichaceae* and species *Parvimonas micra* are associated with decreased LC risk
Seixas [45]	BALF	LC, COPD and ILD	LC (n = 8) vs. COPD (n = 7) vs. ILD (n = 10)	16S V4	No significant changes in diversity between cancer and non-cancer	*Streptococcus* and *Prevotella* are associated with LC *Haemophilus* is associated with COPD
Zheng [22]	BALF	NSCLC	Patients (n = 32) vs. non-cancer controls (n = 15)	16S	Decreased diversity in NSCLC	LC: increased *Lactobacillus rossiae*, *Burkholderia mallei* and *Bacteroides pyogenes*; decreased *Paenibacillus odorifer*, *Pseudomonas entomophila* and *Magnetospirillum gryphiswaldense*
Zhang [46]	Sputum; stool	Metastatic NSCLC	Patients (n = 75) at baseline and during immune checkpoint inhibitors treatment	16S	α-diversity between the gut and respiratory microbiota is not related Only increased α-diversity in the gut is associated with better treatment outcomes	*Streptococcus* in sputum as a biomarker for good treatment response
Dumont-Leblond [47]	Tumor	NSCLC	Tumor tissues vs. adjacent normal tissue from 29 patients	16S V3–V4	Higher β diversity differences among different patients than tissues from the same patient. Higher α-diversity in tumor tissues	LC has an increased abundance of pathogenic and pro-inflammatory bacteria: *Escherichia-Shigella*, *Faecalibacterium*, *Pseudomonas*, *unclassified Enterobacteriaceae*, *Alloprevotella* and *Brevundimonas* High *Phascolarctobacterium* in LUSC
Ma [48]	Tumor	LUAD as SSN or SN	Tumor tissues vs. adjacent normal tissue (n = 10 pairs for SSN; n = 25 pairs for SN)	16S V3–V4	SSN has higher microbiome richness and diversity Tumor and normal tissues have similar diversity and richness	Increased *Actinobacteria*, *Proteobacteria*, *Parvibaculales*, *Parvibaculaceae*, *Parvibaculum*, *Renibacterium* and *Ancylobacter*; and decreased *Firmicutes*, *Bacteroidetes* and *Lactobacillus* in LUAD
Leng [49]	Tumor; sputum	LC	Tumor tissues vs. adjacent normal tissue (n = 31 pairs); sputum from NSCLC patients (n = 17) vs. cancer-free smoker controls (n = 10)	Droplet digital PCR for 25 NSCLC-associated bacterial genera	NA	Enrichment of *Acidovorax*, *Streptococus* and *Veillonella* in sputum of LUSC Enrichment of *Capnocytophaga* in sputum of LUAD
Druzhinin [50]	Sputum	LC	Patients (n = 66) vs. healthy controls (n = 62); all male	16S V3–V4	Decreased β diversity in LC patients	Increased *Streptococcus*, *Bacillus*, *Gemella* and *Haemophilus* in LC patients Chromosomal aberration frequency is positively associated with increased *Bacteroides*, *Lachnoanaerobaculum*, *Porphyromonas*, *Mycoplasma* and *Fusobacterium*; and decreased *Granulicatella*. Micronuclei frequency is negatively associated with increased *Megasphaera* and *Selenomonas bovis*
Hosgood [51]	Oral rinse	LC	Patients (n = 114) vs. healthy controls (n = 114)	Metagenomic shotgun sequencing	Individuals with lower α-diversity had an increased risk of lung cancer No significant changes in β-diversity	Decreased risk of LC: a higher abundance of *Spirochaetia* and *Bacteroidetes* Increased risk of LC: *Bacilli* class and *Lactobacillales* order
Tsay [52]	Lower airway brushing; buccal brushing	LC	Patients (n = 83)	16S V4	α-diversity is similar across different stages of NSCLC. Higher α-diversity in lower airways than in upper airways	*Veillonella parvula* is associated with LC progression, IL-17 expression and the activation of the immune checkpoint Increased *Moraxella*, *Fusobacterium*, *Pseudomonas* and *Haemophilus*; and decreased *Actinomycetales* in advanced LC *Streptococcus*, *Prevotella* and *Veillonella* enrichment is related to poor prognosis
Zhuo [53]	BALF	LC	From cancerous lung and the contralateral non-cancerous lung (n = 50)	16S V3–V4	No significant changes in α- and β-diversity	Increased risk of LC: genera *Weissella* and *Spiroplasma* Decreased risk of LC: phylum Bacteroidetes (class Bacteroidia and order Bacteroidales)
Kovaleva [54]	Tumor	NSCLC	Tumor tissues vs. adjacent normal tissue (n = 89)	16S V3–V4	Tumor tissues have similar α-diversity, but reduce overall bacterial load	High bacterial load with increased iNOS expression is a favorable prognostic factor; High bacterial load with increased FOXP3^+^ cells is associated with poor prognosis Increased *Propionibacterium* is associated with lower iNOS expression
Cheng [55]	BALF	LC	Patients (n = 32) vs. benign pulmonary diseases (n = 22)	16S V3–V4	Similar richness and evenness in LC	*TM7-3*, *Gemmiger*, *Capnocytophaga*, *Sediminibacterium*, *Blautia* and *Oscillospira* are enriched in LC
Mao [56]	Tumor	LC	Tumor tissues vs. adjacent normal tissue (n = 55)	16S V3–V4	Reduced α-diversity in LC; but no significant changes in β-diversity	*Propionibacterium* is significantly reduced in tumor tissues Other reduced genera include: unclassified *Comamonadaceae*, unclassified *Enterobacteriaceae*, *Rhodobacter*, *Psychrobacter*, *Phormidium*, *Propionibacterium*, *Microbacterium* and *Finegoldia*
Bello [57]	Bronchial biopsy; saliva	Central LC	Patients (n = 25): saliva and biopsies of affected and contralateral bronchi vs. healthy controls (n = 16): saliva and single bronchi biopsy	16S V3–V4	The diversity of salivary sample is comparable in patients and controls	*Streptococcus* has dominated in both affected and contralateral bronchi of patients *Pseudomonas* is dominated in control Increased abundance of *Streptococcus*, *Rothia*, *Gemella* and *Lactobacillus* in patients’ saliva
Druzhinin [58]	Sputum	LC	Patients (n = 17) vs. healthy control (n = 17)	16S V3–V4	No significant differences in α-diversity	Increased genera *Haemophilus* and *Bergeyella*; and decreased genera *Atopobium*, *Stomatobaculum*, *Treponema* and *Porphyromonas* in LC patients Chromosomal aberration frequency is negatively associated with the genus *Atopobium* and positively associated with the species *Alloprevotella*
Wong [59]	Tumor	LC	Tumor tissues vs. adjacent normal tissue (n = 497 for LUAD and 433 for LUSC)	TCGA	NA	The LC-associated microbiome is age and gender-specific *Escherichia coli* str. K-12 substrain W3110 is associated with the survival of aged LUAD patients
Reinhold [21]	Tumor; PO swab; BALF	LC	Patients undergoing surgery (n = 13)	16S V3–V4	Decreased α-diversity in the upper airways	High *Prevotella*, *Veillonella* and *Streptococcus* in the upper airways and BALF High *Pseudomonas*, *Propionibacteria*, *Proteobacteria* and *Actinobacteria* in lung cancer tissues
Bingula [60]	Saliva; BAL (from excised lobe); tumor	NSCLC	saliva, BAL, peritumoral tissues, tumor tissues and adjacent normal tissue from 18 patients	16S V3–V4	Unique β-diversity of BAL Diversity varies depending on lobe location	Tissue samples: dominated by Phylum *Proteobacteria* BAL: dominated by class *Clostridia* Saliva: dominated by class *Bacilli*
Patnaik [61]	Saliva; BALF; tumor	Early recurrentNSCLC	Pre-surgery saliva and BALF; tumor tissues and adjacent normal tissue from 48 patients undergoing surgery	16S	Higher diversity in saliva and BALF; Tumor tissues and adjacent normal tissue have similar diversity	Recurrence is associated with increased genus *Delftia* and decreased *Bifidobacterium* in saliva; as well as increased *Staphylococcus* and decreased *Bacillus* and *Anaerobacillus* in tumor tissues
Ekanayake [62]	BALF; PO swab	LC and BRS	Patients (n = 20 for LC and n = 20 for BRS) vs. healthy controls (n = 20)	16S V3–V4	Increased diversity in patients	*Enterococcus faecalis*, *Corynebacterium tuberculostearicum* and *Keratinibaculum paraultunense* are LC-specific
Huang [63]	Bronchial washing fluid; sputum	LC	Bronchial washing fluid (n = 40) and sputum (n = 52) from LC patients	16S V3–V4	No significant difference in α- and β- diversity between LUAD and LUSC	All from Bronchial washing fluid samples: Genera *Veillonell*, *Megasphaera*, *Actinomyces* and *Arthrobacter* are enriched in LUAD without metastasis Genera *Capnocytophaga* and *Rothia* are enriched in LUSC with metastasis *Streptococcus* is decreased in LUAD upon metastasis *Veillonella* and *Rothia* are increased in LUSC upon metastasis
Jin [64]	BALF	LC	Patients (n = 91) vs. nonmalignant pulmonary diseases (n = 29) vs. healthy controls (n = 30); a validation cohort of 85 patients	Metagenomics	Diversity and richness are reduced in LC patients β-diversity is different between LC patients and healthy controls	*Haemophilus influenzae* shows the greatest difference between LC patients and healthy controls
Gomes [65]	BALF	LC	Patients (n = 49) vs. healthy controls (n = 54)	16S V3-V6	LUSC has a higher diversity than LUAD	Biomarkers for LUAD: *Acinetobacter*, *Propionibacterium*, *Phenylobacterium*, *Brevundimonas* and *Staphylococcus* Biomarkers for LUSC: *Enterobacter*, *Serratia*, *Klebsiella*, *Kluyvera*, *Morganella*, *Achromobacter* and *Capnocytophaga*
Ren [66]	Tumor	LUAD as GGN	Tumor tissues (n = 10) vs. adjacent normal tissue (n = 5)	Whole genome sequencing	High β diversity variation among patients	No significant differences in microbiome compositions between GGNs and normal tissues (except LUAD)
Zhang [67]	Saliva	NSCLC	Patients (n = 39) vs. healthy controls (n = 20)	16S V1-V2	A higher richness and lower diversity in NSCLC patients	In NSCLC: increased *Veillonella*, *Streptococcus*, *Lautropia*, *Leptotrichia*, *Rothia* and *Aggregatibacter*; and decreased *Prevotella_7*, *Fusobacterium*, *Porphyromonas*, *Alloprevotella*, *Prevotella*, *Bacteroides* and *Faecalibacterium* *Veillonella* is positively associated with the Neutrophil-lymphocyte ratio *Streptococcus* is negatively associated with the lymphocyte-monocyte ratio
Wang [68]	Saliva; BALF	PBC	Patients (n = 51) vs. healthy controls (n = 15)	16S V4	Patients have lower diversity in both saliva and BALF samples	*Treponema* (in saliva) and *Filifactor* (in both saliva and BALF) are potential biomarkers for LC
Hosgood [69]	Sputum	LC	Patients (n = 45) vs. healthy controls (n = 45)	16S V1-V2	Lower α-diversity is associated with an increased risk of LC	Decreased relative abundance of Fusobacteria is a risk factor for LC
Peters [70]	Tumor	NSCLC	Tumor tissues vs. remote normal tissue (n = 19 pairs)	16S V4	Tumor tissues have reduced richness and diversity	Increased *Koribacteraceae*; and decreased *Bacteroidaceae*, *Lachnospiraceae* and *Ruminococcaceae* in normal tissues are associated with a better survival outcome
Yang [71]	Saliva	LC	Patients (n = 75) vs. healthy controls (n = 172); all female	16S V1-V2	Tumor tissues have reduced richness and diversity	Increased *Sphingomonas* and *Blastomonas* in LC patients
Liu [72]	Tumor	LC	LC-only (n = 11) vs. emphysema-only (n = 10) vs. both LC and emphysema (n = 19); all heavy smokers	16S V4	The emphysema-only group has a lower diversity	LC vs. emphysema-only: decreased *Proteobacteria* (primarily the genera *Acinetobacter* and *Acidovorax*); and increased *Firmicutes* (*Streptococcus*) and *Bacteroidetes* (*Prevotella*)
Greathouse [73]	Tumor	LC	Patients (n = 143) vs. healthy controls (n = 33) TCGA was used as a validation cohort	16S V3-V5	Control tissues have lower α-diversity	*Acidovorax*, *Klebsiella*, *Rhodoferax* and *Anaerococcus* are enriched in LUSC only
Tsay [74]	Lower airway brushing; buccal brushing	LC	Patients (n = 39) vs. non-cancer patients (n = 36) vs. healthy controls (n = 10)	16S V4	No differences (α- and β-diversity) in buccal samples Significant changes in β-diversity in Lower airway samples between LC and non-cancer/healthy controls	*Streptococcus* and *Veillonella* are highly enriched in the lower airways of LC patients and are associated with ERK and PI3K signaling pathway activation
Liu [75]	Bronchial specimen brushing	LC	Diseased lung and paired contralateral healthy lung (n = 24 pairs) vs. healthy controls (n = 8)	16S V3–V4	α-diversity reduces from healthy site to noncancerous to cancerous site	Genera *Streptococcus* and *Neisseria* are significantly more abundant in LC Genera *Staphylococcus* and *Dialister* are significantly more abundant in healthy controls
Cameron [76]	Sputum	LC	Patients (n = 4) vs. non-cancer controls (n = 6)	16S	No significant changes in α-diversity	*Streptococcus viridans* and *Granulicatella adiacens* are significantly increased in LC patients
Lee [77]	BALF	LC	Patients (n = 20) vs. benign diseases (n = 8)	16S V1-V3	Increased diversity in LC	Phyla *Firmicutes* and *TM7* are significantly increased in LC patients
Yu [23]	Tumor	LC	Tumor tissues (n = 31) vs. remote normal tissue (n = 165)	16S V3–V4	α-diversity is increased with environmental exposures, residence population, smoking and disease history LC has reduced diversity	Biomarkers for advanced LC: Genus *Thermus* Biomarkers for LC metastasis: Genus *Legionella*
Yan [78]	Saliva	LC	Patients (n = 10 for LUAD and n = 10 for LUSC) vs. healthy controls (n = 10)	16S V3 and V6	NA	*Capnocytophaga* and *Veillonella* are promising biomarkers for LUSC The abundance of *Capnocytophaga*, *Selenomonas*, *Veillonella* and *Neisseria* in saliva is significantly changed in LC patients
Hosgood [79]	Sputum; oral rinse	LC	Patients (n = 8) vs. healthy controls (n = 8)	16S V1-V2	The diversity between LC and control is similar in buccal samples, but significantly different in sputum	*Granulicatella*, *Abiotrophia* and *Streptococcus* are enriched in the sputum of LC patients

To have a complete collection of lung cancer-associated microbiome studies, the search term in PubMed is (“lung cancer” [Title/Abstract] OR “lung neoplasm” [Title/Abstract] OR “pulmonary neoplasm” [Title/Abstract] OR “pulmonary cancer” [Title/Abstract] OR “lung adenocarcinoma” [Title/Abstract] OR “pulmonary adenocarcinoma” [Title/Abstract] OR “lung squamous cell carcinoma” [Title/Abstract] OR “squamous cell lung carcinoma” [Title/Abstract] OR “lung large cell carcinoma” [Title/Abstract] OR “large cell lung carcinoma” [Title/Abstract] OR “lung small cell carcinoma” [Title/Abstract] OR “small cell lung carcinoma” [Title/Abstract]) AND (“microbiome” [Title/Abstract] OR “Metagenome” [Title/Abstract] OR “microbiota” [Title/Abstract] OR “microbe” [Title/Abstract]). Meta-analysis, reviews and studies about gut microbiome only are excluded. Only profilings of the microbiome in the oral cavity and respiratory tract were selected, and 60 studies were included (as of 2022, Oct 14). LC: lung cancer; RM: recurrent/metastasis; LUAD: lung adenocarcinoma; LUSC: lung squamous cell carcinoma; NSCLC: non-small cell lung cancer; COPD: chronic obstructive pulmonary disease; ILD: interstitial lung disease; BAL: bronchoalveolar lavage; BALF: bronchoalveolar lavage fluid; GGO: ground-glass opacity; sMPLC: synchronous multiple primary lung cancer; SSN or SN: subsolid nodules or solid nodules; PO: posterior oropharynx; BRS: bronchiectasis; PBC: primary bronchogenic carcinoma; GGN: ground-glass nodules. * For 16S sequencing, some studies failed to provide the hypervariable regions they sequenced.

Microbiome richness and diversity are two important parameters for characterizing microbial communities as microorganisms behave and function as communities. Therefore, two microbiome diversities, α-diversity and β-diversity, are often assessed. The α-diversity is the structure of a microbial community concerning its richness (i.e., number of taxa) and/or evenness (i.e., distributions of abundances of these taxa). On the contrary, β-diversity compares the microbiome’s composition between samples. In most cases, a healthy microbiome has higher α-diversity. Indeed, many studies reported decreased diversity or richness in the lung cancer microbiome compared to controls (Table 1). For example, in a study comprising 162 non-small cell lung cancer (NSCLC) patients, the α-diversity of NSCLC tissues was significantly lower than its adjacent normal lung tissues. Moreover, the α-diversity keeps decreasing as the disease progresses [29]. Similarly, although recurrent or metastatic lung cancer has a similar α-diversity as non-recurrent or metastatic lung cancer, its microbial richness is significantly reduced [24]. Regarding anti-PD-1 therapy, reduced lung cancer microbiome diversity is also related to worse clinical outcomes and poorer patient survival [41]. On the contrary, an almost equal number of studies suggested that lung cancers have a similar (or increased) microbial diversity compared to various controls (e.g., tissues from healthy volunteers, adjacent paracancerous normal tissues or normal tissues distant from tumors). For instance, Dong et al. [39] analyzed lung cancer tissues and normal control tissues that were distant from the tumors of a large cohort of 143 patients. They reported that the α-diversity of lung cancers and distant normal lung tissues is not significantly different (although there are significant differences in β-diversity between both groups). Similarly, in two independent studies with two distinct cohorts of 89 non-small cell lung cancer (NSCLC) patients [54] and 35 lung adenocarcinomas (LUAD) [48], the microbiomes of lung cancer tissues and their adjacent normal tissues exhibit similar α-diversity as well. Therefore, there are substantial inconsistencies across these studies, significantly increasing the difficulty in identifying lung cancer-specific biomarkers.

### 2.2. Potential Reasons for the Few Commonalities across Studies

Such a lack of consensus across studies may be related to multiple factors, including sampling techniques, sequencing methods, the choice of controls, and most importantly, environmental and host variations. Firstly, as aforementioned, the low microbial biomass of the lung renders it very sensitive to contamination when handling the sample. However, in most studies, the microbiome of lung cancer tissues is compared to that of the adjacent paracancerous “normal” lung tissues. These samples experience similar background contamination during processing; therefore, unique lung cancer-resident bacteria can still be identified. This suggests that potential contamination may not be the primary cause of the few commonalities across studies.

Secondly, although most studies used16S rRNA gene sequencing for bacteria identification, different variable regions of 16S rRNA were sequenced in these studies (Table 1). There are nine hypervariable regions (V1–V9) in bacterial 16S rRNA genes, and V1, V2, V3, V4, and/or V5 were mostly sequenced for detecting lung microbiomes. However, no single region can differentiate all bacteria, and each region has its relative advantages in identifying different bacterial species [80]. It would be ideal to sequence multiple regions of 16S rRNA genes, as Wu et al. [26] performed in their study. Other than 16S rRNA sequencing, targeted pathogen sequencing [32,36,49,81], whole-genome sequencing (WGS) [66] or RNA-seq (which may be obtained from TCGA database) [24,73,82,83] were used to identify lung cancer microbiomes. The application of targeted pathogen sequencing may miss some novel bacteria species, and the ability to detect microbes using RNA-seq or WGS dramatically depends on the sequencing the depth of the experiments. Therefore, it is inevitable that dissimilarities exist across studies.

Thirdly, various control samples were used for comparison in these studies. As introduced earlier, tissues from healthy volunteers or benign lung diseases, adjacent paracancerous normal tissues, normal tissues distant from tumors or contralateral noncancerous tissues were used as baseline references (Table 1). However, the microbiome’s diversity varies among these controls, and the previous review indicated that this might be the primary cause of variations among the studies [84]. Indeed, Greathouse et al. [73] reported that the α-diversity is lower in normal lungs compared to adjacent paracancerous normal tissues. However, in another study with a similar design, Liu et al. [75] observed that the α-diversity decreased from healthy controls to the contralateral noncancerous lung of lung cancer patients to lung cancer tissues. Moreover, contradictory findings were provided for studies using adjacent paracancerous normal tissues as controls (refer to the examples provided previously [29,48,54]). These findings indicate that the choice of the control is not the underlying reason for the lack of consensus observed in lung microbiome profiling.

Fourthly and most importantly, as the lung is under constant environmental exposure, environmental factors would be the primary cause of variations in the lung microbiome. This could also explain the inconsistent results observed in various controls. Indeed, in their analysis of the impact of smoking and indoor air pollution (i.e., coal burning) on the lung cancer microbiome, Chen et al. [19] found that the pollutants significantly decreased the biodiversity of lung tissue-specific microbes, especially in paracancerous normal tissues. Pollutant-detoxication microbes (such as *Sphingomonas* and *Sphingopyxis*) are highly enriched in the lungs to protect their lungs under such conditions. Similarly, Dong et al. [39] reported that polycyclic aromatic hydrocarbon (PAH)-degrading microbes, *Massilia* and *Sphingobacterium*, are more abundant in lung cancer tissues of smokers than non-smokers. Hosgood et al. [79] reported similar findings on coal-burning pollutions as well. Interestingly, the abundance of another PAH-degrading microbe, *Acidovorax*, is cancer subtype-specific. It is only highly enriched in the lung squamous cell carcinoma (LUSC) tissues (and not LUAD tissues) of smokers [39,73]. In a large cohort analysis of lung microbiome in 165 noncancerous lung tissues from lung cancer patients, the α-diversity of the lung microbiome is dependent on various environmental factors: air pollutions/air particulate matters, the population density of residential area, and smoking or lung disease histories [23]. Notably, even tobacco smoking and electronic cigarette usage also result in different microbial communities in the lungs [85]. These findings indicate that the lung microbiome is sensitive to even very subtle environmental changes. Researchers should consider all these identified and unidentified environmental factors when analyzing lung microbiome data.

Finally, studies have suggested high β-diversity differences in lung cancer microbiome among patients [66]. Host or patient variations are also significant contributors to the dissimilarities of different lung microbiome profilings, although some host variations are closely related to environmental factors. As aforementioned, LUSC and LUAD have different enriched specific tumor-resident bacteria (e.g., *Acidovorax*) [39,73]. This is further confirmed by another analysis of 497 LUAD and 433 LUSC patients, which indicated that both LUAD and LUSC tumor tissues contain unique microbiomes compared to their adjacent paracancerous tumor tissues [59]. Moreover, the authors reported that such lung cancer microbiomes are also age- and gender-specific. In older LUAD patients (both male and female), *Escherichia coli* strain K-12 substrain W3110 dysregulation is a promising prognostic marker for patient survival. On the contrary, *Pseudomonas putida* strain KT2440 is uniquely enriched in young LUSC male patients. Similarly, Chen et al. [19] also reported that females have a higher α-diversity than males in both tumor tissues and adjacent paracancerous normal tissues. Other than tumor subtypes, gender and age, gene/pathway mutation-specific microbes are also identified. For example, lung cancers with EGFR mutation are associated with the absence of *Pseudomonas. aeruginosa* [43], the increased abundance of *Serratia marcescens* or the decreased abundance of *Haemophilus parainfluenzae* [36]. However, it remains to be determined whether such gene/pathway mutations lead to specific microbe enrichments or whether specific microbes drive certain gene mutations. Nevertheless, these specific microbes can still be used as potential prognostic biomarkers.

In conclusion, the lung microbiome is very sensitive to external variations. Studies have already begun to identify lung cancer-associated microbes in patients with specific conditions (e.g., heavy smokers [72], never smokers [51] or females only [71]). However, larger cohorts with much more complex patient stratifications are still required to thoroughly examine the association of specific microbes with lung cancers in patients under various conditions.

## 3. Airway or Respiratory Tract Microbiome and Lung Cancers

As lung biopsy is hard to access and obtaining “true health” lung tissues in most cases is unethical, numerous studies with respect to lung microbiomes are based on bronchoalveolar lavage, bronchial washing, sputum or oral samples. There were concerns about the potential contamination of the lung microbiome from airway/oral bacteria [86]. However, as introduced earlier, the lung microbiome is closely related to (and maybe even originated from) the airway’s microbiome [20,22], which also reflects the spatial relationship of different sampling sites [61]. Therefore, the airway’s microbiome can still represent lung-resident microbial communities. While variations (even significant variations) exist among microbiomes at different sites of airways/mouth [21,22,26,60,63,73,81], studies still identified lung cancer-specific bacteria using these samples (Table 1). Unfortunately, a lack of consensus still exists among these studies on airway microbiomes due to similar reasons introduced for lung microbiomes. Nevertheless, the ease of access still renders airway/oral microbes ideal candidates for lung cancer biomarkers. The most commonly examined samples were obtained from the oral cavity or bronchoalveolar lavage fluid (BALF).

The oral cavity harbors the second largest microbial community in our body; therefore, oral microbiomes possess a rich repertoire of microbes for biomarker identification. Oral samples can be obtained via oral wash [28,51,73] or saliva [38,57,60,61,67,68,71,78,85]. Other than oral samples, another easily accessible sampling site/biospecimen is sputum [25,27,37,42,46,49,50,58,60,63,69,76,87] or posterior oropharynx [21,62]; the sputum is the second most-used sample for lung cancer-associated microbiome studies. In an analysis of potential biomarkers for NSCLC metastasis using sputum and gut microbiomes, Lu et al. [42] suggested that several microbial biomarkers are shared between the sputum and gut, and the prediction power of sputum microbial biomarkers is similar to that of the combination of the sputum and gut. This further supports the use of sputum microbiome in lung cancer prognosis.

Other than the mouth and upper respiratory tract, the lower respiratory tract (other than the lung) is another popular (and mainly studied) sampling site for lung cancer-associated microbiome studies. Such samples can be acquired chiefly via BALF, although bronchial washing fluid and broncho epithelial brushing were adopted as well (refer to Table 1 as there are too many studies). The BALF sample has several unique advantages, including the relative ease of access, proximity to lung tissues, lower invasiveness and less ethical problems (especially when compared to healthy lung tissue samples). Moreover, although the lower respiratory tract may possess a unique microbiome (compared to the microbiome in other sites) [57,60,61], several independent studies reported that BALF or bronchial washing fluid is one of the most representative biospecimens for lung cancer-associated microbiome study among different sampling sites of the respiratory tract. Huang et al. [63] analyzed bronchial washing fluids and sputum samples from lung cancer patients and found that the microbiome of bronchial washing fluids was more similar to that of lung cancer tissues. They further observed that microbial biomarkers observed in bronchial washing fluid were more significantly associated with the different stages and subtypes of lung cancers (compared to those of sputum samples), indicating that bronchial fluid samples reflect lung cancer-specific microbes better. In addition, another study reported that the lung cancer-specific microbes detected in BALF mostly cover those identified in lung cancer tissues [32], further indicating that bronchial samples are an ideal alternative to lung tissues for lung cancer-associated microbiome study.

## 4. Frequently Altered Bacterial Genera in Lung Cancer Patients

While different lung microbiome profilings provided inconsistent results, certain bacteria are still frequently identified as lung cancer-specific (regardless of sampling sites), and their roles in lung cancer tumorigenesis have been elucidated. Therefore, these bacteria are promising lung cancer biomarkers and will be discussed in the following sections.

### 4.1. Veillonella

Although being a genus highly dominated in the airways and oral cavity [21,33], *Veillonella* in the respiratory tract or mouth is still perhaps one of the most identified microbial lung cancer biomarkers. Currently, there are 10 studies indicating that the abundance of *Veillonella* at various sites of the respiratory tract (e.g., sputum, saliva and BALF) is highly associated with lung cancers (or poor prognosis and distant metastasis of lung cancers, regardless of tumor subtypes), even the controls used were different [21,34,38,49,52,63,67,74,77,78]. For example, by analyzing airway brushing samples from lung cancer patients, non-cancer patients and healthy controls, Tsay et al. [74] found that oral taxa *Veillonella* is only highly enriched in lung cancer patients. The same group further analyzed the microbiome of lower airway brushing, transcriptomic data and the clinical data of 83 lung cancer patients (some transcriptomic data and clinical data were missing) and observed that *Veillonella* is highly enriched in patients with poor prognosis and tumor progression [52]. Similarly, an analysis of BALF samples [77] or salivary samples [78] demonstrated that *Veillonella* could be a potential biomarker of lung cancers. Interestingly, although *Veillonella* is the dominant genus in the induced sputum of either NSCLC patients or patients with chronic obstructive pulmonary disease (COPD), its abundance is decreased in patients with both NSCLC and COPD [27]. The authors proposed that the latter cases might be the conditions of cancer progression driven by COPD-induced inflammation, and the decreased *Veillonella* results from stress tolerance.

Due to the prevalence of *Veillonella* in lung cancer, the potential mechanism of *Veillonella* in lung cancer progression has also been studied. It has been shown that the upregulation of extracellular signal-regulated kinase (ERK) and phosphoinositide 3-kinase (PI3K) signaling pathways in lower airway transcriptomes of lung cancer patients is significantly associated with the enrichment of *Veillonella* in the same location [74]. Moreover, the in vitro co-culture of the *Veillonella* supernatant with airway epithelial cells leads to the activation of the PI3K pathway and the upregulation of inflammasome-related genes (e.g., IL-17) [74]. It has been shown that the PI3K pathway activation in the airway epithelium contributes to lung cancer initiation and progression [88,89], suggesting *Veillonella’s* potential carcinogenic role in lung cancer. In the subsequent study from the same group, the authors further confirmed this association using a KP mice model of lung cancer (with conditional activatable oncogenic *Kras* and *Trp53* mutation, the mouse homolog of TP53). The intra-tracheal inoculation of *Veillonella parvula* to KP mice leads to lung dysbiosis and induces the strongest lower airway inflammation among all potential microbial biomarkers [52]. This results from the increased recruitment of Th17 and neutrophils, enhanced IL-17 production and an elevated expression of PD-1 in T cells [52]. Another study also reported that the systemic inflammation marker, neutrophil-lymphocyte ratio, is positively associated with the abundance of *Veillonella* in the saliva of NSCLC patients [67]. Zeng et al. [34] reported that *Veillonella* significantly promotes lung cancer progression in C57BL/6 mice as well. It should be noted that the association between PD-1^+^ T cells and *Veillonella* may make *Veillonella* a potential predictor of anti-PD-1 immunotherapy responses, as *Veillonella dispar* is dominated in the BALF of lung cancer patients with high PD-L1 expression [40]. These patients are also responders to immunotherapy. In conclusion, *Veillonella* can promote lung cancer progression by creating a pro-cancer immune microenvironment.

Notably, besides directly regulating host cells, *Veillonella* can also modulate the microbial community. In the murine model with CT26 colon carcinoma cells, *Veillonella* can significantly increase the abundance of pro-inflammatory bacteria *Pseudomonas aeruginosa* in tumor tissues. Increased *Pseudomonas aeruginosa* leads to increased blood TNF-α and poor survival outcomes [90]. In lung cancer, *Pseudomonas* is enriched in BALF [22], lower airway tract [52], cancer tissues [47,54] or the sputum of patients with distant metastasis as well [42]. The increased abundance of *Pseudomonas* in lung cancers may result from enriched *Veillonella*. Therefore, *Veillonella* may potentially modulate other lung cancer-associated microbes (e.g., *Pseudomonas*) and indirectly contribute to lung cancer progression.

### 4.2. Prevotella

Similarly to *Veillonella*, *Prevotella* is a genus that is highly enriched in airways [21,33]. There are currently nine studies showing *Prevotella* as a potential microbial biomarker for lung cancers [21,29,32,34,38,45,52,72,74]. Interestingly, *Prevotella* seems to be highly associated with *Veillonella*, as five studies observed that both *Prevotella* and *Veillonella* are enriched in lung cancer patients (although the correlation between *Prevotella* and lung cancer is not as significant as that for *Veillonella*) [21,34,38,52,74]. A typical healthy and balanced oral or lung microbiome consists of genera, including *Streptococcus*, *Neisseria Prevotella*, *Veillonella*, *Porphyromonas* and *Fusobacterium* [91]. It is possible that pathogenic events during lung cancer progression affect both *Prevotella* and *Veillonella* at the same time. However, the possibility of opportunistic infections cannot be excluded. Similarly to *Veillonella*, the co-culture of *Prevotella* with airway epithelial cells also leads to the upregulation of ERK and PI3K signaling pathways [74]. Therefore, *Veillonella* and *Prevotella* likely play similar roles in lung cancer progression.

### 4.3. Streptococcus

Perhaps *Streptococcus* is the most commonly detected genus across all lung cancer-associated microbiome profilings (using various samples). Over 20 studies demonstrate that changes in *Streptococcus* abundance are related to lung cancer (Table 1). While inconsistency exists, most of these studies reported that the abundance of *Streptococcus* positively correlates with lung cancer progression. Mechanistically, *Streptococcus* leads to the upregulation of ERK and PI3K signaling pathways in airway epithelial cells, which is the same as *Prevotella* and *Veillonella* [74]. In addition, it has been proposed that *Streptococcus mitis* can induce inflammation, Th17 activation and PD-L1 expression, leading to tumorigenesis (similarly to *Veillonella*) [57]. However, such chronic lung inflammation is potentially caused by lung dysbiosis; whether *Streptococcus* plays a vital role in it remains to be determined. Moreover, as *Streptococcus* is one of the most abundant genera in the respiratory tract [91], the enrichment of *Streptococcus* in lung cancer patients may result from opportunistic infection. Its presence in lung tissue may be a result of microaspirations from the oral cavity [57]. There are similar concerns for the other aforementioned dominant genera in the respiratory tract (i.e., *Prevotella* and *Veillonella*). However, the contributing roles of *Veillonella* in tumor progression have been elucidated, suggesting that it is not an opportunistic pathogen. While the precise role of *Streptococcus* in lung cancer remains to be determined, the increased abundance of *Streptococcus* can still be potentially used as a biomarker for immune dysregulation and lung cancer.

### 4.4. Acidovorax

Other than *Veillonella*, another commonly identified microbial lung cancer biomarker with potential mechanisms studied is *Acidovorax.* As introduced earlier, *Acidovorax* is a PAH-degrading microbe specifically associated with the LUSC of smokers [39,73]. Besides these two studies, other studies also reported that *Acidovorax* is enriched in LUSC tissues [49], and another targeted analysis of *Acidovorax* revealed that the abundance of *Acidovorax* is significantly elevated in LUSC patients with COPD or patients with LUSC relapse after surgery compared to patients with other conditions [92]. Interestingly, these studies also reported that the presence of *Acidovorax* is highly associated with TP53 mutations in lung cancers [39,73,92].

As a PAH-degrading bacterium, the enrichment of *Acidovorax* in the respiratory tract may result from environmental pressure from smoking or pollution. It is widely known that smoking directly leads to immune dysfunction and damages epithelial cells [93]. *Acidovorax* can protect these damaged cells by degrading toxic smoke compounds. Therefore, these damaged cells survive and contribute to potential tumorigenesis. Moreover, these pollutant-detoxication microbes have metabolic advantages and may form biofilms. Biofilm can potentially damage epithelial layers, leading to further bacterial invasion, DNA damage and chronic inflammation [94].

Furthermore, preliminary data suggested that *Acidovorax temperans* suppress both innate and adaptive immunity [95]. The phagocytosis of M2 macrophages is decreased after *Acidovorax temperans* engulfment, and these M2 macrophages can suppress T cell function via CD47-SIRPα immune checkpoints. Therefore, the nasal delivery of *Acidovorax temperans* accelerates lung tumorigenesis in the KPC murine models of LUAD driven by lung-specific Ad-Cre-activated *Kras* and *Trp53* mutations [95]. The same group also observed that *Acidovorax temperans’* exposure in murine lung cancer models increases pro-inflammatory cells (mostly neutrophils), CD4^+^ T cells and CD4^–^ CD8^–^ T cells (including RORγt^+^ IL17^+^ T cells). These results suggested that *Acidovorax temperans* can reshape the immune microenvironment of lung cancer and promote tumorigenesis [96].

### 4.5. Haemophilus

There are 11 independent lung cancer-associated microbiome sequencing studies [21,25,32,36,38,42,50,52,58,64,81] and one traditional culture-dependent study [97] demonstrating the association between *Haemophilus* enrichment (at various sites) and lung cancer (although such an association was not as significant as those between lung cancer and *Veillonella*). The commonly identified species of *Haemophilus* include *Haemophilus parainfluenzae* and *Haemophilus influenzae*, but *Haemophilus haemolyticus* is reported as well. However, the predictive value of *Haemophilus* abundance in lung cancer may require further validation, as there are studies indicating contradictory results: *Haemophilus* is only enriched in paracancerous normal tissue [49] or in the BALF of patients with COPD [45]. Such inconsistent observations are most likely a result of the fact that the *Haemophilus* genus is a ubiquitous bacterium that can be found in the respiratory tract of nearly 80% of healthy individuals [98]. Therefore, *Haemophilus* may be an opportunistic pathogen in lung cancer patients, as their immune systems are compromised.

### 4.6. Capnocytophaga

Most *Capnocytophaga* bacteria are normal bacteria commonly found in the oral cavity or oropharyngeal tracts of humans. However, under immune-compromised conditions, such as lung cancer, *Capnocytophaga* can be an opportunistic pathogen. Indeed, six studies reported an elevated *Capnocytophaga* abundance in the salivary, sputum, BALF and tumor tissues of lung cancer patients [19,44,49,55,65,78]. However, although the enrichment of *Capnocytophaga* in lung cancer may result from opportunistic infection, studies also reported that *Capnocytophaga* contributes to lung abscess formation [99]. Therefore, it is still possible that *Capnocytophaga* induces long-term inflammation, ultimately leading to tumor formation.

### 4.7. Other Commonly Identified Lung Cancer-Associated Microbes

The association between *Acinetobacter* and lung cancers has been reported extensively. However, there is enormous inconsistency across the studies: Five studies indicated that *Acinetobacter* was elevated in lung cancer patients [29,43,65,76,81], while four other studies reported that the abundance of *Acinetobacter* decreased in the same condition [27,36,71,72]. Such a lack of consensus occurs at the species level as well. This suggests that the variation in *Acinetobacter* abundances in lung cancer patients may result from microbiome dysbiosis, and *Acinetobacter* does not play an essential role in tumorigenesis. Similarly, contradictory results have been observed for *Staphylococcus* (Table 1). A further analysis should be performed at the species level (where many studies failed to do so). In addition, several studies have also reported an increased abundance of *Rothia* in lung cancer patients (Table 1). However, few papers suggested it can be a promising biomarker.

## 5. Conclusions

**Figure 1:**
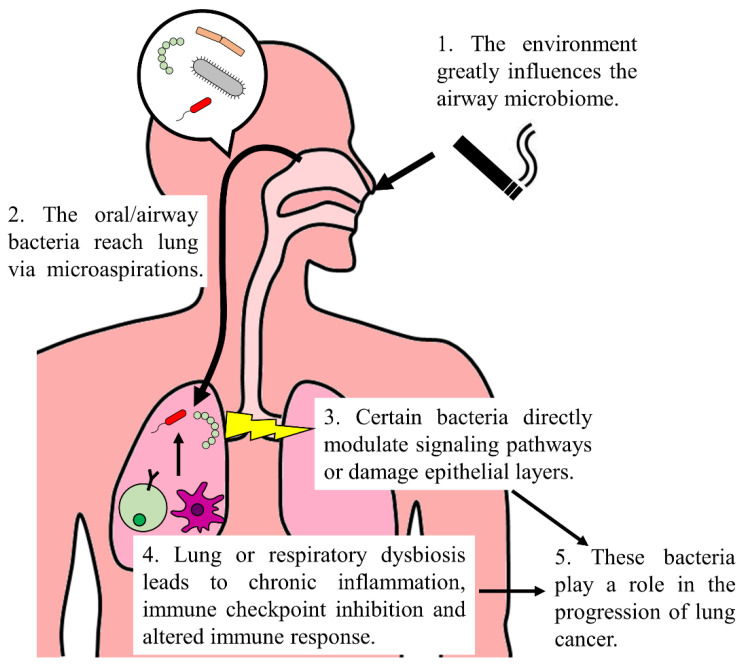
Involvement of the microbiome in lung cancer. The environmental factors (e.g., smoking) are the primary cause of variations in oral and airway microbiomes. These bacteria reach lung via microaspirations. Certain bacteria, like *Veillonella* and *Acidovorax*, can directly upregulate ERK and PI3K signaling pathways or damage epithelial cells. Moreover, lung or respiratory dysbiosis reshapes immune response, leading to aggravated Th17 and neutrophil responses, chronic inflammation and elevated levels of immune checkpoint molecules. Taken together, these microorganisms contribute to lung cancer development.

According to the WHO, lung cancer is the second most common cancer and the global leading cause of cancer-related death [14]. While the precise mechanisms require further elucidation, the microbiome undoubtedly plays an essential role in lung cancer initiation and progression (Figure 1). Therefore, numerous researchers have identified potential microbial biomarkers for lung cancers. However, a lack of consensus still exists across these studies. Some critical issues in this field still need to be addressed: Firstly, there are technical problems and variations in the studies’ designs. The collection of lung cancer-related microbiomes is challenging, and they are sensitive to contamination. The detection methods and controls used in each study vary as well. Initiating an international, multi-center, extensive cohort analysis (with standardized methodology) to identify potential microbial biomarkers of lung cancer thoroughly would be ideal. However, only one international, multi-center, extensive cohort analysis is not enough, because the second issue that needs addressing is the environmental influence on the respiratory tract’s microbiome. Therefore, regional analyses are still required to establish a local panel of biomarkers. Thirdly, lung/respiratory microbiome dysbiosis (instead of a specific bacterial species) seems to be a driving force in lung cancer formation. With the development of multi-omics and spatial transcriptomics analysis, the exact interaction between different microbes and cell types can be revealed. Lastly, the roles of other microbes (i.e., fungi and viruses, other than bacteria) in lung cancer remain unknown. Recently, a pan-cancer mycobiome analysis (using TCGA data) revealed that *Blastomyces* are enriched in lung cancer tissues [100]. A preliminary assessment of virome in the cancer tissue of LUAD also showed that lung cancer might possess unique virus compositions [101]. Hence, fungi and viruses can also serve as promising biomarkers for lung cancer, and further profilings are required.

While there are still some critical problems and substantial inconsistencies in all lung cancer-associated microbiomes across the studies, a consensus is still reached with respect to the association between certain bacteria genera (such as *Veillonella*, *Acidovorax*, *Prevotella*, *Streptococcus*, *Haemophilus* and *Capnocytophage*) and lung cancer. While not all lung cancer-associated microbiome profilings detect the enrichment of these genera in lung cancers, the abundance of these genera is only increased in lung cancer patients. Therefore, these genera can serve as risk factors for lung cancer. Due to environmental variations and patient dissimilarities, obtaining globally accepted microbial biomarkers for lung cancer may be impossible. However, the microbiome studies of the respiratory tract still identified numerous microbes as risk factors. This can significantly help the diagnosis and prognosis of lung cancer patients.
